# Supplementary material for: Deep learning-based screening for locomotive syndrome using single-camera walking video: Development and validation study
Source: PLOS Digit Health. 2024 Nov 26;3(11):e0000668. doi: 10.1371/journal.pdig.0000668 (PMC11593753; doi:10.1371/journal.pdig.0000668)
Supplement: S1 Appendix — (DOCX) [file pdig.0000668.s001.docx]

**S1 Appendix**

| GLFS-25 |  |
| --- | --- |
| ﻿Q1 | Pain in neck or upper limbs |
| Q2 | Pain in back or buttocks |
| Q3 | Pain or numbness in lower limbs |
| Q4 | Painful to move body in daily life |
| Q5 | Difficulty getting up from bed or lying down |
| Q6 | Difficulty standing up from a chair |
| Q7 | Difficulty walking inside the house |
| Q8 | Difficulty putting on and taking off a shirt |
| Q9 | Difficulty putting on and taking off pants |
| Q10 | Difficulty using the toilet |
| Q11 | Difficulty washing the body in the bath |
| Q12 | Difficulty going up and down stairs |
| Q13 | Difficulty walking briskly |
| Q14 | Difficulty keeping yourself neat |
| Q15 | Walking distance without rest |
| Q16 | Difficulty going out to visit neighbors |
| Q17 | Difficulty carrying objects weighing approximately 2 kg |
| Q18 | Difficulty using public transportation |
| Q19 | Difficulty doing simple tasks and housework |
| Q20 | Difficulty doing load-bearing tasks and housework |
| Q21 | Difficulty performing sports activity |
| Q22 | Refrain from meeting friends |
| Q23 | Refrain from joining social activities |
| Q24 | Fall-related anxiety |
| Q25 | Anxiety about being unable to walk in the future |
