# Supplementary figures and images for: Deep learning-based screening for locomotive syndrome using single-camera walking video: Development and validation study
Source: PLOS Digit Health. 2024 Nov 26;3(11):e0000668. doi: 10.1371/journal.pdig.0000668 (PMC11593753; doi:10.1371/journal.pdig.0000668)

**S2 Appendix**

**
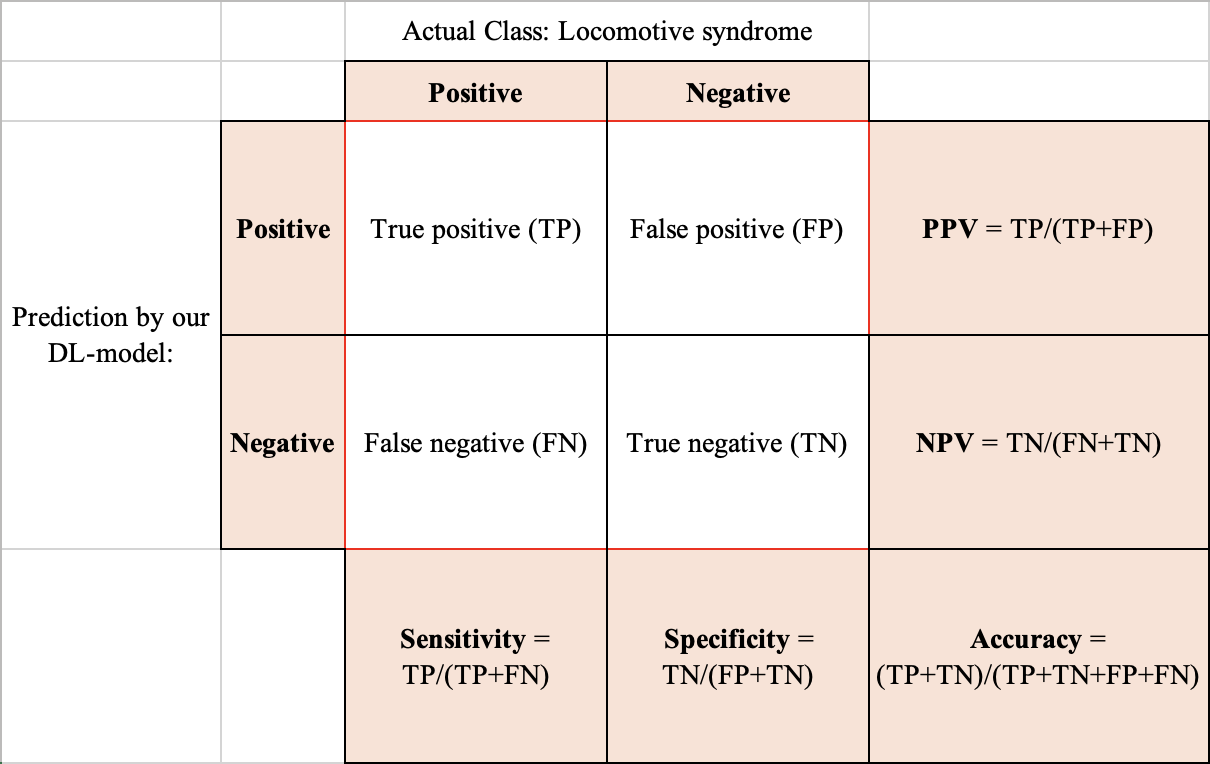
**

Supplement: S2 Appendix — (DOCX) [file pdig.0000668.s002.docx]
