# Supplementary material for: Deep learning-based screening for locomotive syndrome using single-camera walking video: Development and validation study
Source: PLOS Digit Health. 2024 Nov 26;3(11):e0000668. doi: 10.1371/journal.pdig.0000668 (PMC11593753; doi:10.1371/journal.pdig.0000668)
Supplement: S3 Appendix — (DOCX) [file pdig.0000668.s003.docx]

**S3 Appendix**

|  | participant ID | age (y) | sex | height (cm) | LS class |
| --- | --- | --- | --- | --- | --- |
| Detailed characteristics of the participants in the model creation group | 1 | 50 | F | 160 | 2 |
|  | 2 | 81 | M | 170 | 3 |
|  | 3 | 79 | F | 145 | 3 |
|  | 4 | 78 | M | 163 | 3 |
|  | 5 | 79 | F | 149 | 2 |
|  | 6 | 72 | M | 165 | 2 |
|  | 7 | 82 | M | 162 | 3 |
|  | 8 | 83 | M | 170 | 3 |
|  | 9 | 75 | F | 157 | 3 |
|  | 10 | 81 | F | 151 | 3 |
|  | 11 | 74 | M | 170 | 2 |
|  | 12 | 80 | F | 150 | 2 |
|  | 13 | 88 | M | 167 | 2 |
|  | 14 | 59 | M | 169 | 1 |
|  | 15 | 82 | M | 161 | 3 |
|  | 16 | 51 | F | 156 | 1 |
|  | 17 | 53 | F | 165 | 0 |
|  | 18 | 64 | M | 169 | 0 |
|  | 19 | 26 | F | 153 | 0 |
|  | 20 | 24 | F | 156 | 0 |
|  | 21 | 25 | F | 168 | 1 |
|  | 22 | 25 | F | 160 | 0 |
|  | 23 | 49 | F | 163 | 0 |
|  | 24 | 44 | M | 174 | 0 |
|  | 25 | 75 | F | 158 | 3 |
|  | 26 | 86 | M | 163 | 3 |
|  | 27 | 72 | M | 170 | 1 |
|  | 28 | 40 | F | 160 | 3 |
|  | 29 | 22 | F | 160 | 0 |
|  | 30 | 21 | F | 164 | 1 |
|  | 31 | 79 | F | 146 | 3 |
|  | 32 | 65 | F | 153 | 3 |
|  | 33 | 76 | F | 149 | 3 |
|  | 34 | 78 | M | 162 | 3 |
|  | 35 | 80 | F | 153 | 2 |
|  | 36 | 70 | M | 177 | 1 |
|  | 37 | 68 | F | 157 | 3 |
|  | 38 | 79 | F | 145 | 1 |
|  | 39 | 74 | M | 153 | 2 |
|  | 40 | 85 | F | 144 | 3 |
|  | 41 | 73 | F | 154 | 3 |
|  | 42 | 54 | M | 172 | 1 |
|  | 43 | 58 | F | 154 | 0 |
|  | 44 | 20 | F | 164 | 0 |
|  | 45 | 56 | M | 175 | 0 |
|  | 46 | 46 | M | 175 | 0 |
|  | 47 | 43 | M | 167 | 0 |
|  | 48 | 25 | M | 171 | 0 |
|  | 49 | 62 | F | 153 | 3 |
|  | 50 | 46 | F | 153 | 1 |
|  | 51 | 28 | F | 157 | 0 |
|  | 52 | 42 | F | 147 | 0 |
|  | 53 | 66 | F | 160 | 1 |
|  | 54 | 71 | F | 144 | 1 |
|  | 55 | 67 | F | 154 | 1 |
|  | 56 | 78 | M | 170 | 3 |
|  | 57 | 83 | F | 153 | 3 |
|  | 58 | 66 | F | 157 | 0 |
|  | 59 | 69 | F | 158 | 1 |
|  | 60 | 71 | F | 151 | 1 |
|  | 61 | 81 | M | 167 | 1 |
|  | 62 | 89 | F | 139 | 3 |
|  | 63 | 69 | F | 138 | 3 |
|  | 64 | 70 | F | 145 | 3 |
|  | 65 | 80 | M | 173 | 2 |
|  | 66 | 71 | F | 144 | 0 |
