# Supplementary material for: Deep learning-based screening for locomotive syndrome using single-camera walking video: Development and validation study
Source: PLOS Digit Health. 2024 Nov 26;3(11):e0000668. doi: 10.1371/journal.pdig.0000668 (PMC11593753; doi:10.1371/journal.pdig.0000668)
Supplement: S4 Appendix — (DOCX) [file pdig.0000668.s004.docx]

**S4 Appendix**

|  | participant ID | age (y) | sex | height (cm) | LS class |
| --- | --- | --- | --- | --- | --- |
| Detailed characteristics of the participants in the external validation group | 1 | 56 | M | 164 | 0 |
|  | 2 | 76 | M | 165 | 1 |
|  | 3 | 72 | F | 159 | 1 |
|  | 4 | 59 | F | 161 | 1 |
|  | 5 | 63 | F | 148 | 1 |
|  | 6 | 62 | F | 158 | 1 |
|  | 7 | 71 | F | 145 | 0 |
|  | 8 | 61 | F | 148 | 0 |
|  | 9 | 44 | M | 180 | 1 |
|  | 10 | 76 | F | 148 | 3 |
|  | 11 | 67 | F | 158 | 1 |
|  | 12 | 57 | F | 153 | 1 |
|  | 13 | 69 | M | 163 | 0 |
|  | 14 | 40 | F | 160 | 0 |
|  | 15 | 72 | F | 153 | 1 |
|  | 16 | 41 | M | 172 | 1 |
|  | 17 | 50 | F | 165 | 1 |
|  | 18 | 75 | F | 155 | 3 |
|  | 19 | 76 | M | 170 | 3 |
|  | 20 | 55 | M | 174 | 1 |
|  | 21 | 51 | F | 155 | 1 |
|  | 22 | 71 | F | 154 | 0 |
|  | 23 | 60 | F | 156 | 0 |
|  | 24 | 65 | F | 154 | 0 |
|  | 25 | 49 | F | 156 | 1 |
|  | 26 | 74 | F | 149 | 1 |
|  | 27 | 77 | M | 165 | 2 |
|  | 28 | 55 | F | 151 | 0 |
|  | 29 | 32 | M | 170 | 0 |
|  | 30 | 34 | F | 168 | 0 |
|  | 31 | 78 | M | 158 | 2 |
|  | 32 | 74 | F | 157 | 1 |
|  | 33 | 88 | M | 156 | 1 |
|  | 34 | 66 | F | 163 | 1 |
|  | 35 | 78 | F | 153 | 1 |
|  | 36 | 69 | F | 159 | 0 |
|  | 37 | 81 | F | 153 | 2 |
|  | 38 | 70 | M | 176 | 1 |
|  | 39 | 72 | M | 163 | 1 |
|  | 40 | 34 | M | 165 | 0 |
|  | 41 | 55 | F | 150 | 0 |
|  | 42 | 74 | F | 151 | 0 |
|  | 43 | 69 | F | 152 | 1 |
|  | 44 | 85 | F | 145 | 1 |
|  | 45 | 77 | F | 148 | 2 |
|  | 46 | 65 | M | 163 | 1 |
|  | 47 | 73 | F | 146 | 0 |
|  | 48 | 73 | M | 171 | 1 |
|  | 49 | 94 | M | 145 | 1 |
|  | 50 | 81 | F | 156 | 1 |
|  | 51 | 77 | F | 161 | 3 |
|  | 52 | 76 | F | 162 | 1 |
|  | 53 | 75 | F | 153 | 0 |
|  | 54 | 56 | F | 156 | 0 |
|  | 55 | 24 | F | 163 | 0 |
|  | 56 | 73 | M | 166 | 1 |
|  | 57 | 28 | F | 165 | 1 |
|  | 58 | 71 | F | 149 | 1 |
|  | 59 | 71 | F | 146 | 3 |
|  | 60 | 44 | F | 165 | 1 |
|  | 61 | 39 | M | 172 | 1 |
|  | 62 | 83 | F | 148 | 1 |
|  | 63 | 85 | F | 151 | 1 |
|  | 64 | 37 | M | 170 | 0 |
|  | 65 | 21 | M | 169 | 0 |
