# Supplementary material for: Deep learning-based screening for locomotive syndrome using single-camera walking video: Development and validation study
Source: PLOS Digit Health. 2024 Nov 26;3(11):e0000668. doi: 10.1371/journal.pdig.0000668 (PMC11593753; doi:10.1371/journal.pdig.0000668)
Supplement: Attachment — Submitted filename: response_to_the_reviewers-v23.docx [file pdig.0000668.s005.docx]

**Responses to the comments of Reviewers**

We would like to thank the reviewers for their helpful constructive comments which helped us significantly improve our manuscript. We have addressed all the issues indicated in the review report, hoping that the revised version meets the journal publication requirement.

Our detailed responses to the comments of Reviewer #1 are presented below.

**Reviewer #1:**

Prevalence of Locomotor Syndrome:

1. The manuscript lacks information on the prevalence of locomotor syndrome, both globally and within the studied population. It is important to provide this context to understand the significance and applicability of your results. It is necessary to include references to the global prevalence of locomotor syndrome and, if possible, provide data on the prevalence within the specific population you studied. This information is crucial for interpreting your findings accurately.

We appreciate the reviewer’s suggestion. We briefly described the prevalence of locomotive syndrome in the world and in Japan in the introduction part of our revised manuscript (lines 114-115 and lines 121-123 on page 6) and added references regarding the prevalence of locomotive syndrome (Reference number 3 and 10 on page 26 and 228).

2. It is necessary to include the formula to calculate positive and negative predictive values, specificity, etc

Following the reviewer’s advice, we included the table that describes the formula to calculate PPV, NPV, sensitivity, specificity, and accuracy in the method section of our revised manuscript (lines 495-497 on page 23), and S2 Appendix in the supporting information.

Equivalence of Training and Validation Groups:

3. There is a noticeable imbalance between the training and validation groups in terms of the number of patients and their stages of locomotor syndrome. The training group includes 24 patients in stage 3, 9 patients in stage 2, and 15 patients in stage 1. In contrast, the validation group has 5 patients in stage 3, 4 patients in stage 2, and 35 patients in stage 1. This discrepancy can affect the validity and reliability of your model’s performance. Please address this imbalance. Possible solutions include:
   A) Expanding the dataset to ensure more balanced groups.
   B) Providing a detailed justification for the current group composition and discussing its implications on the study’s results.

We appreciate the reviewer for pointing this out. We agree with the reviewer and are in the process of recruiting cases (especially stage-2 and stage-3LS patients) to resolve this imbalance and will evaluate the performance of our updated model in the next research project by eliminating the bias in the training data and also by preparing several unbiased validation cohorts.

In the current study, the subjects are clinically classified into 4 classes, "normal", "stage-1 LS", "stage-2 LS" and "stage-3 LS", based on the results of the LS risk test. However, when these data are used for deep learning model development, the model is trained as a binary classification; the non-LS group, which includes "normal" subjects, and the LS group, which includes stage 1 to 3 LS patients, as shown in Figure 1. In the training dataset, the non-LS group consists of 52 videos and the LS group consists of 134 videos, and the percentage of the number of videos of the LS group is 72% [134/(134+52)]. In the validation cohort dataset, the non-LS group consists of 21 videos and the LS group consists of 44 videos, and the proportion of the LS group is 68% [44/(44+21)], which is comparable to that of the training dataset. Therefore, we believe that there is no significant imbalance in the proportion of the LS group between the training dataset and the validation dataset, although we admit that there is an imbalance within the training dataset (non-LS : LS = 52 : 134, as shown in Figure 1 in our current study, which is desired to be 50 : 50). This imbalance within the training dataset could have a potential negative impact on our current model, possibly leading to increased false positives (or decreased specificity). We have decided to include this point in the Discussion section of our revised manuscript (lines 370-373 on page 18).

Statistical Analysis and Model Validation:

4. Given the imbalance in patient distribution, it is important to discuss how this might impact the model’s performance. Include statistical analyses that account for the differences in group sizes and stages. Consider performing additional validation using more balanced groups if possible.

We agree with the reviewer that it is important to discuss the potential impact of the imbalance. Indeed, we have already identified that there is a significant imbalance between the cohort of the training dataset and the validation cohort. As shown in Table 3 in line 231 (on page 11 and 12) of our revised manuscript, the chi-square test shows that the stage distribution of the LS is significantly different between the two groups. Following the reviewer’s advice, we are planning to evaluate the performance of our current model on a more balanced validation cohort in the future.

5. Discuss any potential biases introduced by the current group compositions and how they were mitigated in your analysis

As we mentioned above, there is no significant imbalance between our training dataset and validation dataset.

Our detailed responses to the comments of Reviewer #3 are presented below.

**Reviewer #3:**

Results:

1. Line 154: Table 1. The n (%) should be placed in the column header instead to prevent repeating information. Additionally, if the female numbers are already presented, why not present the male numbers as well? This would prevent readers from having to perform their own calculations.

According to the reviewer’s advice, we modified Table 1 in line 175 and presented the number of males in our revised Table 1 (on pages 8 and 9).

Discussion:

2. Line 265: How would the “model’s challenge in reliably excluding non-LS cases” affect clinical decisions?
3. Line 286: Similar to my comment above, how would the model’s "tendency towards false positives” affect clinical decisions? Would unnecessary resources be put into these false positive cases? How would this affect the current workflow of clinicians to diagnose LS?

Our model was developed as a screening tool to assist physicians in diagnosing locomotive syndrome as part of their clinical practice. Certainly, our current model is in its developmental stage and tends to generate a high number of false positives, but even when false positives are identified by our model, the benefit to the patient is not compromised because the physician can ultimately make a clinical diagnosis that the false positive case is actually negative. We consider false negatives to be more problematic than false positives, and we do not believe that the high false positive rate of our model is a major problem as a screening tool. In addition, using this model, it is less burdensome for the clinician to preferentially examine patients who test positive after screening with this model, compared to a clinician diagnosing locomotive syndrome in an entire cohort by examining each patient individually. This model does not waste medical resources in clinical practice and obviously improves physician’s workflow.

Methods:

4. The statements below are similar and make the paragraph verbose. Please avoid repeating information, especially if they are in the same paragraph.

Line 382: “ …ensuring compliance with ethical standards and patient safety.”

Line 384: “…reinforcing the ethical integrity and transparency of the research process.”

Line 386: “…confirming adherence to ethical guidelines.”

Following the reviewer’s advice, we modified the sentences in the method section of our revised manuscript (lines 407-408 on page 19).

5. Line 418: “…one to four times”. How did you decide which participant(s) to do one, two, three, or four times? Please elaborate on this.

We apologize for the confusion. In fact, participants were instructed to walk three times when videos were recorded, not four times. In most cases, three videos per participant could be successfully saved and played back, but for technical reasons, some of the videos recorded could not be played back, and in such cases, no more than two videos per participant were employed as a training dataset. Most of the videos could be played back without technical issues, and we did not arbitrarily select videos and employ them as a training dataset. We corrected the simple innocent errors described above and modified the misleading expressions in our revised manuscript (line 439-440 on page 21, lines 445-447 on page 21).

6. Line 418: “from a side view”. Were the video recordings from the left or right side of the participants? From Figure 4, sample images showed were from the right side. Are these consistent for all participants? Please elaborate on this.

We appreciate the reviewer's comments. The video was taken from the participant’s right side. We include this information in the method section of our revised manuscript (line 440 on page 21).

7. It repeats the information mentioned in line 413. Is it referring to the same camera mentioned in line 413? If yes, I do not see the need to repeat this information in the next paragraph. If it is necessary to mention the use of Advance Video Coding (AVC), would it be possible to combine it with line 413 instead? Or remove the repeating information of resolution and fps in line 421.

Line 413: “…with a resolution of 1288 x 964, 30 FPS,”

Line 421: “at a resolution of 1288 x 964 and a frame rate of 30 FPS”.

We appreciate the reviewer's comments and apologize for any redundancy. Based on the reviewer’s advice, we removed the repeating information of resolution and fps in line 421 in our originally submitted manuscript.

8. Paragraph line 454 on OpenPose. Given that the video recordings were from the left or right side of the participants, did you encounter any issue with occlusions or swapping of lower-limb pose estimation? If yes, what were the steps taken to fix these issues? Were there any checks done before inputting to MS-G3D?

In our preliminary study, we observed that the OpenPose program’s posture estimation incorrectly estimated the left and right sides of the legs for subjects wearing black pants, but no other types of estimation errors were observed. Taking advantage of this prior study, we instructed participants to avoid black pants in both the training and validation data cohort in our study and recorded walking videos. Therefore, we did not encounter any issue with swapping of lower-limb pose estimation.

9. Line 460: What do you mean by “converted into graph-structured format”? How is this different from the OpenPose default output of pose estimation?

We apologize for the confusion. The Openpose default output contains the positional coordinates of the keypoints in numerical order but does not include information on which keypoints are connected to which keypoints. On the other hand, the “graph-structured format” consists of the position coordinates of each keypoint as well as the linkage information between keypoints. In MS-G3D, keypoint connectivity information is required for convolution processing in the direction of graph connectivity. This is why we converted the keypoint data obtained from Openpose into graph-structured format.

10. Line 468: "...the gait is classified as indicative of LS; otherwise, it is classified as non-LS." So the model prediction of LS is binary, but in paragraph line 426 on the LS risk test, patients can be classified into multiple stages depending on severity. Please elaborate on why you chose a binary classification instead of replicating the same classification of the LS risk test.

We apologize for the inadequate explanation and description of the points you raised. There are two reasons why we chose a binary classification. The first reason is that we don’t have enough videos of stage-2 LS patients in the training dataset (please see Figure 1) to build a 4-class classification model. The second reason is that we need to develop a screening program that can accurately discriminate between normal subjects (i.e., non-LS participants) and diseased subjects (i.e., LS patients). These are why we developed a binary classification model, not a 4-class classification model.

11. Additionally, if the model prediction is binary, how did Figure 3 come about? What does ‘accuracy’ here refer to? I assume is the model vs doctor diagnosis. Then how did you know when the model classified different LS stages here?

We apologize for the confusion. This bar graph shows the accuracy of the model’s prediction. Specifically, the far-left bar represents the proportion of accurate and inaccurate predictions of the model in LS stage 0 participants. This means that in the validation cohort, about 40% (indicated by a blank area) of LS-stage 0 participants were accurately predicted by our model (i.e. non-LS), while about 60% of them (indicated by a shaded area) were predicted by our model as having LS. Similarly, the far-right bar shows that about 80% of the participants of stage-3 LS subjects were accurately predicted (i.e. as having LS), while 20% of them were predicted to be in the “non-LS” group. This figure does not represent the comparison between the model prediction and the physician diagnosis.

12. Paragraph line 472: What is the inter-reliability between the 6 orthopedic surgeons? Were all orthopedic surgeons unanimous in classifying LS for all 65 walking video clips?

We appreciate the reviewer’s fruitful comments. In our analysis, the Fleiss’ kappa value, which represents the interrater-reliability between the six orthopedic surgeons, is 0.372, suggesting that there is a fair agreement of the interpretation between them (*Landis, J. R.; Koch, G. G. (1977), "The measurement of observer agreement for categorical data", Biometrics, 33 (1): 159–174, doi:10.2307/2529310, JSTOR 2529310, PMID 843571*). In addition, all six surgeons blinded to the clinical information evaluated all 65 walking videos recorded in the validation cohort and judged whether the examinees in the video were in LS condition.

13. Line 488: The chi-square test was mentioned but I did not see any chi-square statistical values reported in the results section.

We used the chi-square test in Table 3 (line 231 on pages 11 and 12 in our revised manuscript) in order to compare accurate and inaccurate groups by DL-model for external validation.

14. Figure 1. The amount of non-LS and LS cases is imbalanced, almost 2.5 times, in model creation. Do you think this would contribute to the model leaning towards false positives?

We appreciate the reviewer’s comment. We admit that the imbalanced training dataset in our current study could have a potential negative impact on our current model, possibly leading to increased false positive cases (or decreased specificity). However, this problem can be eliminated by increasing the minority class (non-LS class in our current study) of the training dataset. We decided to include this point in the discussion section of our revised manuscript (lines 370-373 on page 18).

15. Figure 3, part a. Where did these “40 frames” come from?

We apologize for the confusion we caused. Reviewer #3 wrote "Figure 3, part a", which I believe is a mistake for “Figure 4 panel a”, so we will discuss “Figure 4 panel a” here. The reason for choosing 40 frames is that a gait video of 40 frames (by 30 fps = 1.3333… seconds) typically contains more than one cycle of gait, which allows for a complete representation of the subject's gait characteristics, even if the subject walks very slowly due to some disease. We added a brief description of the reason for choosing 40 frames in the method section of our revised manuscript (lines 478-479 on page 22).
